# Dataset of suppression subtractive hybridization libraries of banana-biostimulant-*Pseudocercospora fijiensis* molecular interaction

**DOI:** 10.1016/j.dib.2019.104557

**Published:** 2019-10-05

**Authors:** Tatiana Paola Chávez-Navarrete, Eduardo Sánchez-Timm, Efrén Santos-Ordóñez

**Affiliations:** a ESPOL Polytechnic University, ESPOL, Centro de Investigaciones Biotecnológicas del Ecuador, Campus Gustavo Galindo, Km. 30.5 vía Perimetral, P.O. Box 09-01-5863, Guayaquil, Ecuador; b ESPOL Polytechnic University, Escuela Superior Politécnica del Litoral, ESPOL, Facultad de Ciencias de la Vida, Campus Gustavo Galindo, Km. 30.5 vía Perimetral, P.O. Box 09-01-5863, Guayaquil, Ecuador

**Keywords:** Plant-biostimulant interaction, Plant-pathogen interaction, Gene expression, SSH-method

## Abstract

Two subtractive cDNA libraries from banana leaves (cultivar ‘Williams’, genotype AAA) after biostimulant application on the leaf (library 1) or the substrate (library 2), with *Pseudocercospora fijiensis* infection were generated. The banana plants were applied first with the biostimulant and later the inoculation of *P. fijiensis* was performed on the leaves after one week. The suppression subtractive hybridization was performed by using as tester the treatments with biostimulant application by sampling banana leaves after two weeks of *P. fijiensis* inoculation, and every two weeks for two months (four time points); while the driver were collected on the same dates on independent banana plants that were only inoculated with *P. fijiensis* (no biostimulant application). The plants were maintained in the greenhouse for the entire assay.

**Table:** Specifications Table

Subject	Agricultural and Biological Sciences (General)
Specific subject area	Molecular Biology interaction between Plant-Biostimulant-Pathogen
Type of data	Figures
How data were acquired	Leaf samples were collected from plants grown in greenhouse for RNA extraction; while the ESTs were generated by developing a library using the suppression subtractive hybridization (SSH) technique. The sequencing was performed commercially (Macrogen, USA).
Data format	Raw data from sequencing. Analysed and Filtered
Parameters for data collection	The developmental stage of the plants was phase II in the greenhouse (banana plants derived from *in vitro* culture after a period of 6–8 weeks in greenhouse with substrate) with at least two expanded leaves. Six biological replicates were collected for each time point and treatment.
Description of data collection	ESTs were obtained by sequencing in Macrogen (USA) directly from the library developed by the SSH technique.
Data source location	Guayaquil , Ecuador , -2.1509699, -79.9536332
Data accessibility	Mendeley Data , https://doi.org/10.17632/sbhmv5ckd9.1 Direct URL to data: data.mendeley.com/datasets/sbhmv5ckd9/1

**Table:** 

**Value of the Data** Molecular interaction between biostimulants and plants associated to a pathogen infection is shown, allowing other researchers to target specific genes related to biostimulants or biofertilizers interactions with plants. The ESTs collected from the interaction provide access to researchers to analyse this information and to compare it with other, or directly target to a specific gene or group of genes. Information related to differentially expressed-genes present in the interaction with the biostimulant is indicated. This could open new questions of how genes are involved in the improvement of the plant fitness and/or (a)biotic stress tolerance.

## 1 Data

The uploaded database was obtained from banana leaves of the cultivar *Musa acuminata* ‘Williams’ (genotype AAA), with the application of a liquid biostimulant and after the infection of *Pseudocercospora fijiensis* to the leaves. The samples collected correspond to the second leaf of each plant, using two different treatments: 1) application of biostimulant before infection of *P. fijiensis* directly to the leaves (L1); and, 2) application of the biostimulant directly to the substrate before *P. fijiensis* infection (L2). For both treatments, the control was the infection of the plant with the *P. fijiensis* without the application of the biostimulant. The SSH libraries were developed independently. This database was analysed by BLAST2Go software [1] with a total of 176 unique sequences for L1 and 116 unique sequences for L2.

**Fig. 1:**
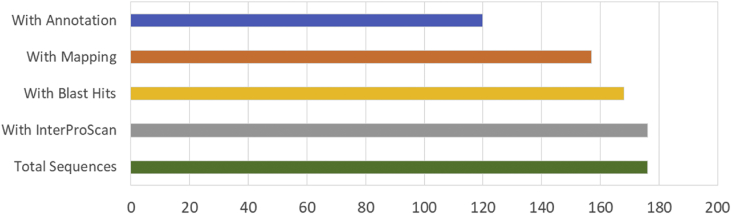
Statistics analysis for L1 using B2Go software. Where the x-axis indicates the total number of sequences obtained in each analysis/process showed in the y-axis.

**Fig. 2:**
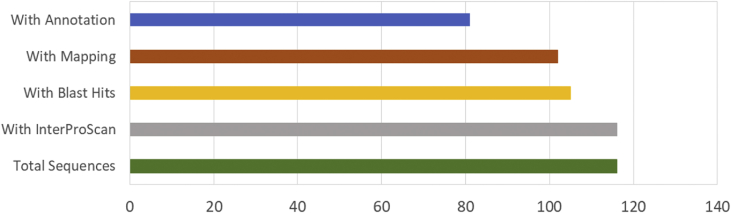
Statistics analysis for L2 using B2Go software. Where the x-axis indicates the total number of sequences obtained in each analysis/process showed in the y-axis.

**Fig. 3:**
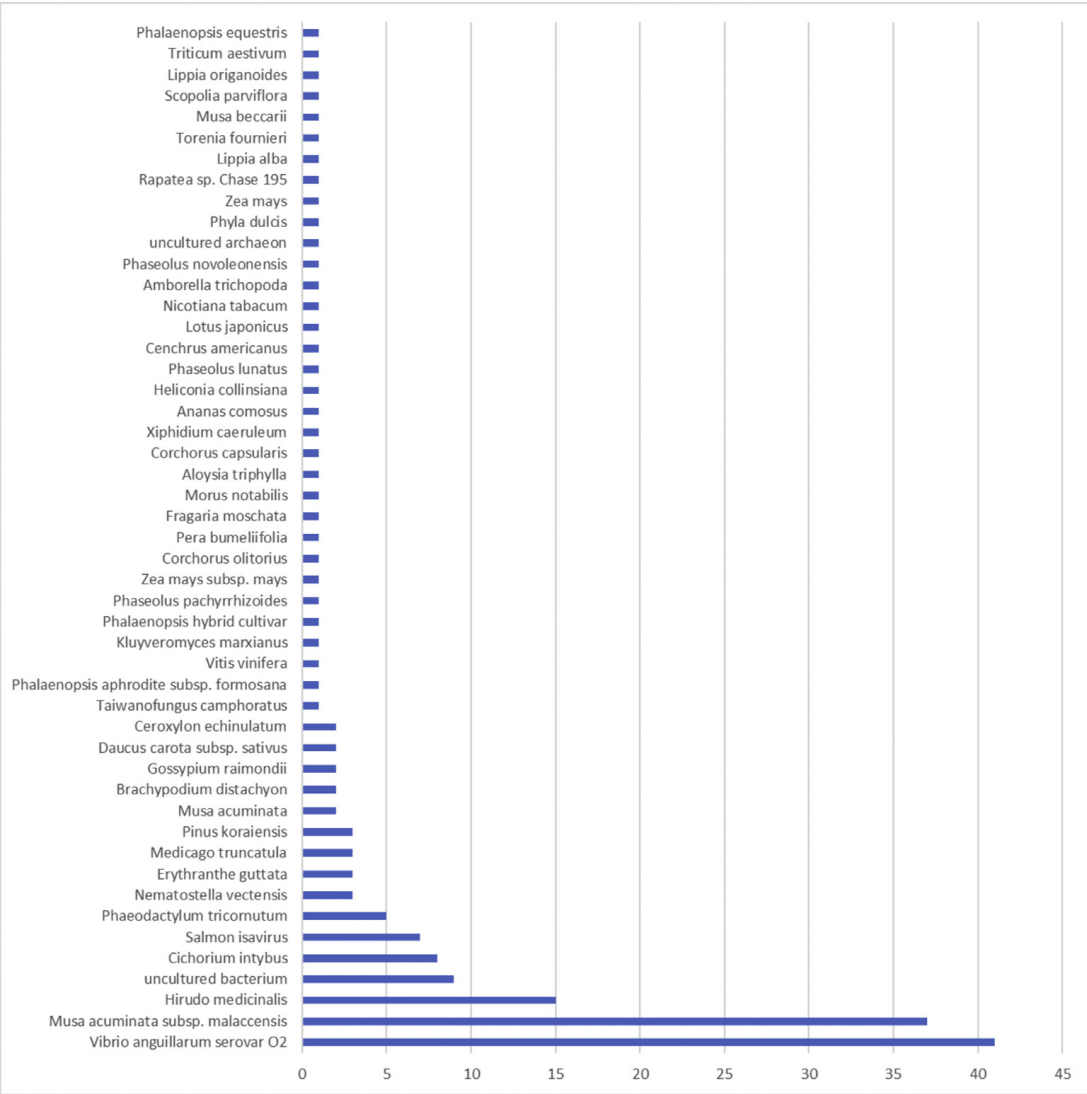
Top hits statistics species by B2Go software for L1. X-axis shows the number of sequences related to the organism described in the y-axis.

**Fig. 4:**
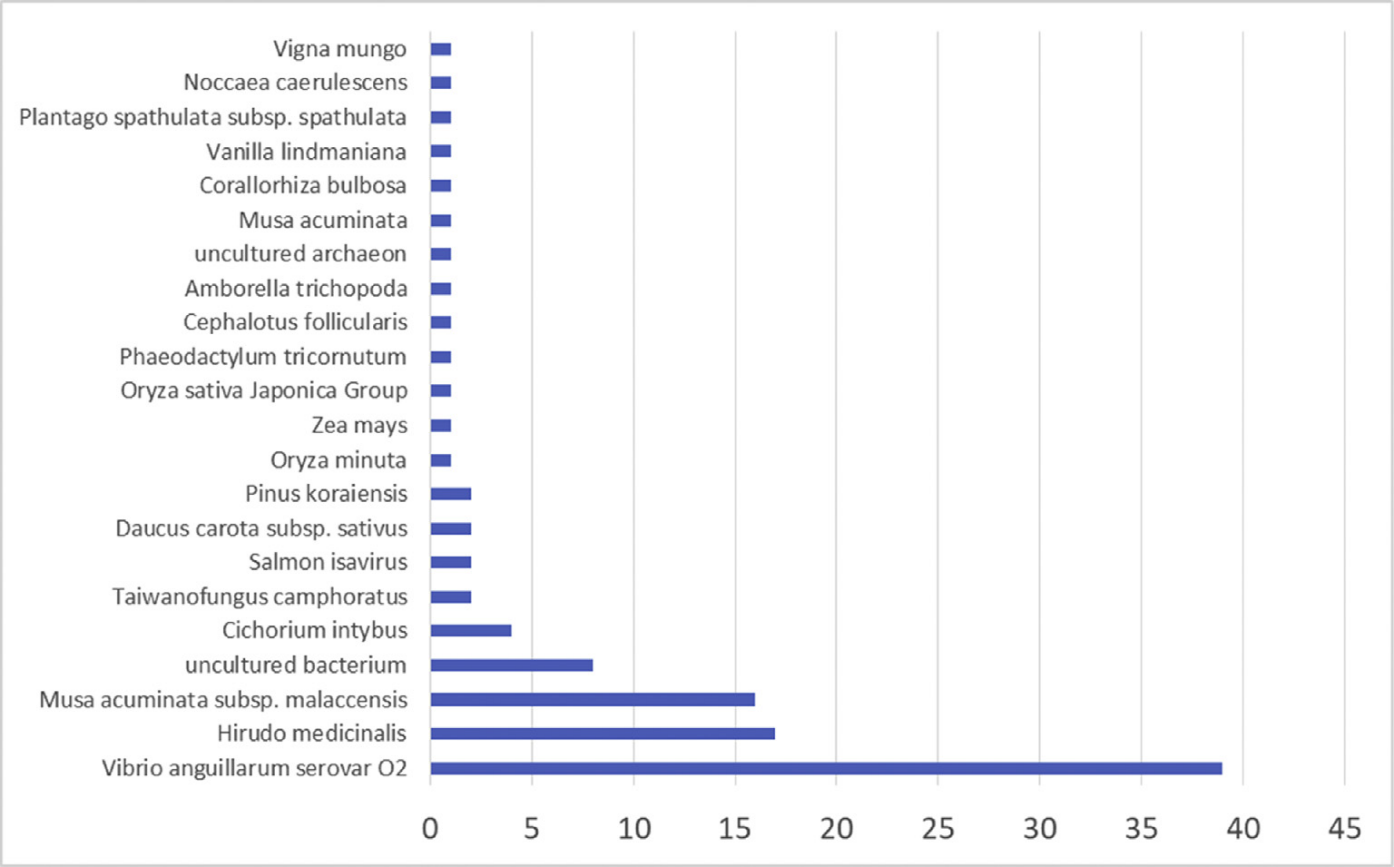
Top hits statistics species by B2Go software for L2. X-axis shows the number of sequences related to the organism described in the y-axis.

**Fig. 5:**
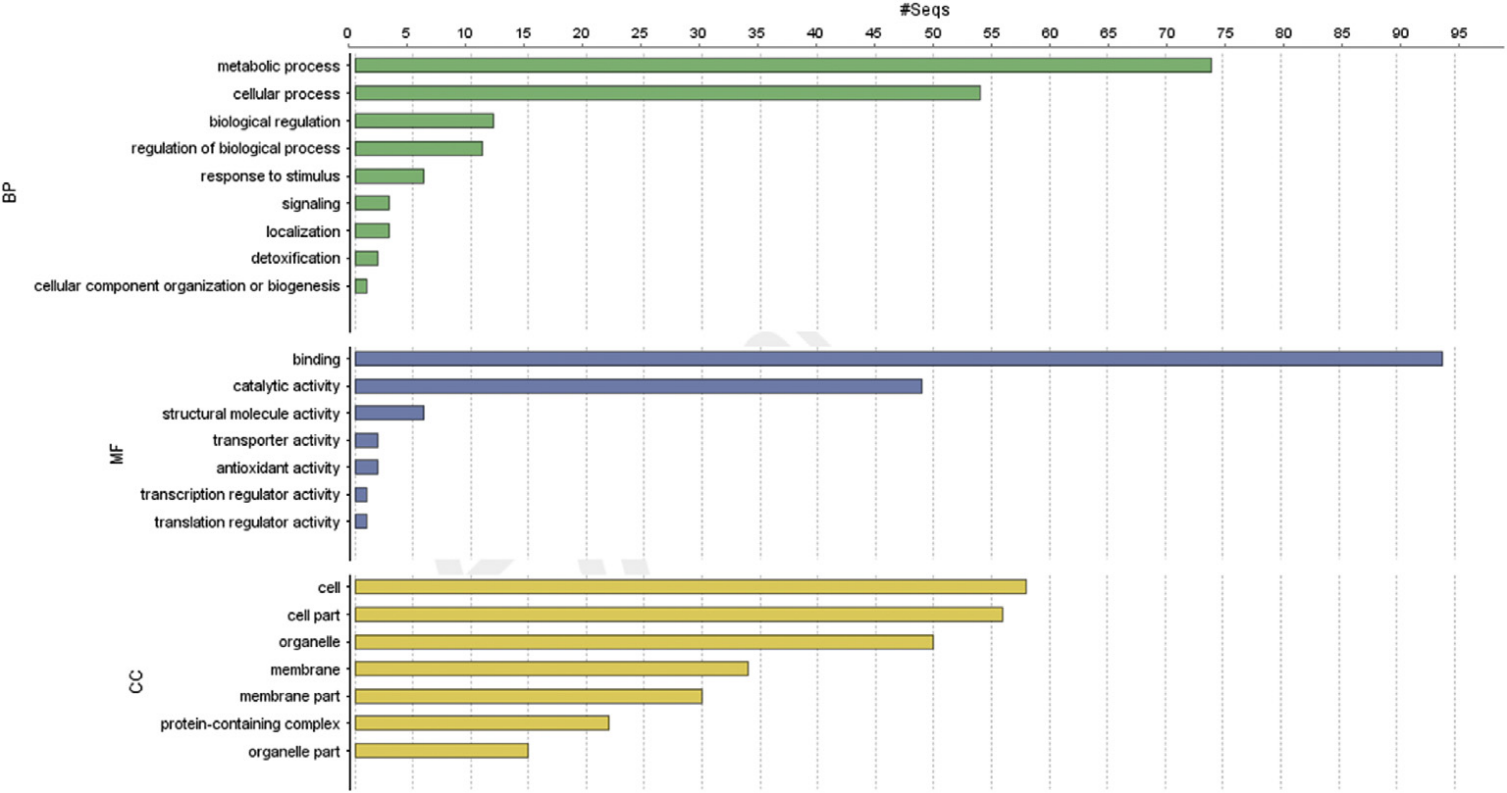
GO distribution by level 2 for the L1 of annotated EST by software B2Go. Where the x-axis represents the number of sequences with hits in CC: Cellular Component; MF: Molecular Function; BP: Biological Process, represented in the y-axis.

**Fig. 6:**
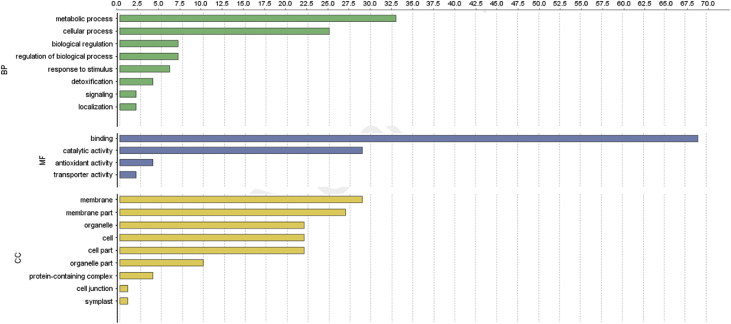
GO distribution by level 2 for the L2 of annotated EST by software B2Go. Where the x-axis represents the number of sequences with hits in CC: Cellular Component; MF: Molecular Function; BP: Biological Process, represented in the y-axis.

**Fig. 7:**
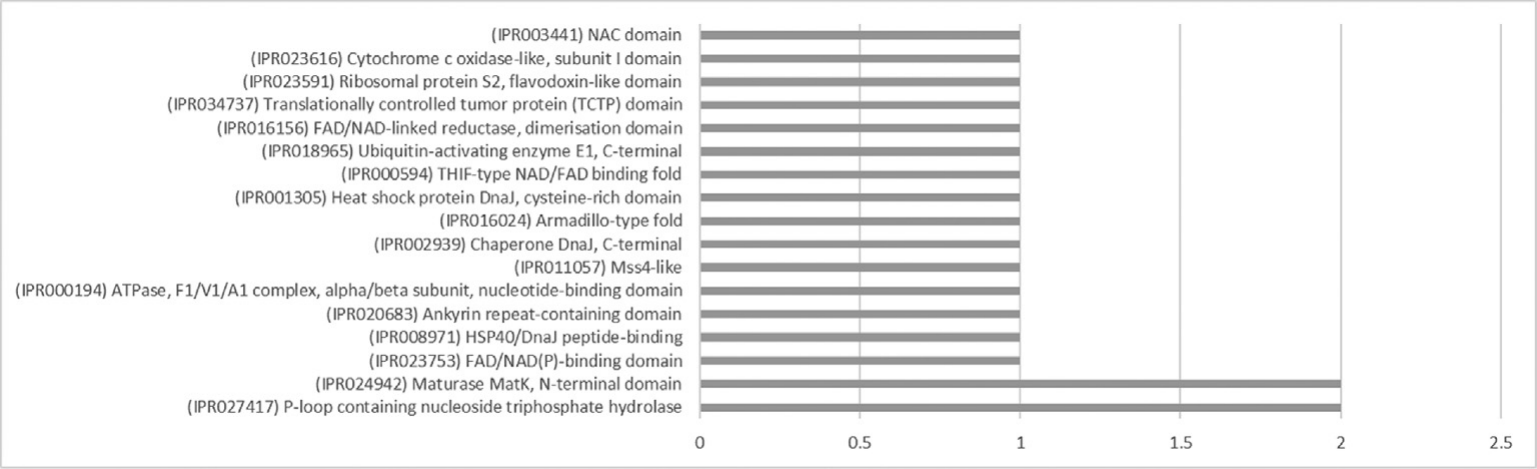
Interpro Domain distribution statistics by B2Go for L1. Where the x-axis represents the number of sequences with hits.

**Fig. 8:**
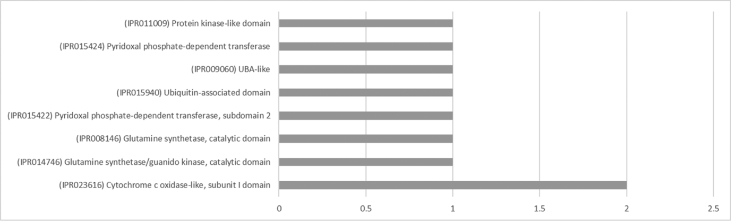
Interpro Domain distribution statistics by B2Go for L2. Where the x-axis represents the number of sequences with hits.

**Fig. 9:**
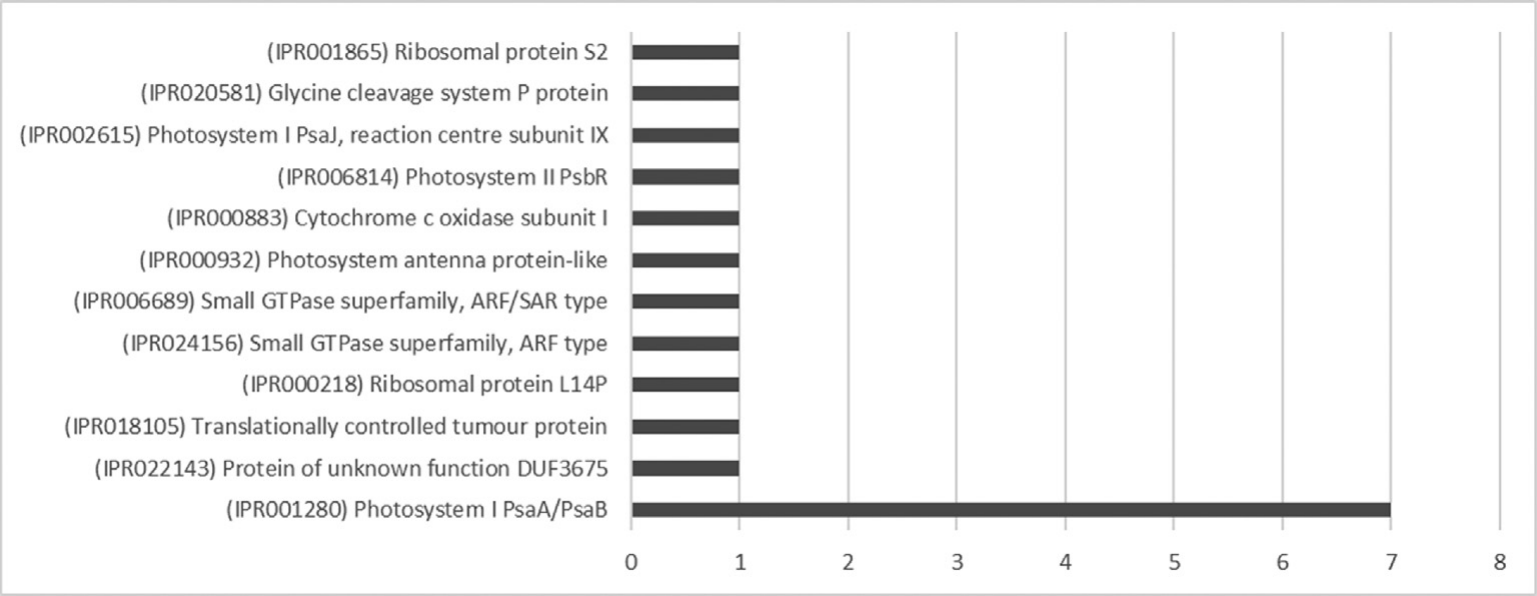
InterproScan Family distribution by B2Go software for L1. Where the x-axis represents the number of sequences with hits.

**Fig. 10:**
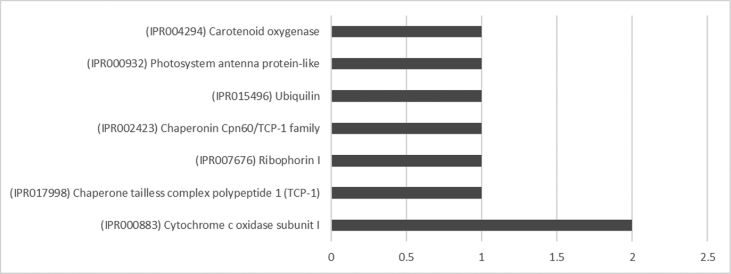
InterproScan family distribution by B2Go Software for L2. Where the x-axis represents the number of sequences with hits.

The result shows that only 120 of 176 had been annotated (Fig. 1) for L1, and 81 of 116 for L2 (Fig. 2). The top hits about plant species for both libraries were *Musa acuminata* subsp. *malaccensis* (Fig. 2, Fig. 3). The gene ontology (GO) distribution showed genes related to molecular function rather than other GO for both libraries (Fig. 4, Fig. 5), but for the L1 the more representative genes were related to binding and some to catalytic activity, structural molecule activity, transporter activity, antioxidant activity, transcription regulatory activity and translation regulatory activity. On the other hand, for the L2 library, the genes found were more related to binding, catalytic activity, antioxidant activity and transport activity. For both libraries the expressed genes had similarity distribution in the GO (see Fig. 6). The Interpro Domain and Interpro Scan Family distributions are indicated (see Fig. 7, Fig. 8, Fig. 9, Fig. 10).

Blastn analysis at the *P. fijiensis* genome in both libraries only showed that two EST clones were similar to genes of *P. fijiensis*. The clones 5A10 and 5D2 showed DNA similarities to the accessions Mycfi2|scaffold_7:3592859-3592966 (2.57E-16) and Mycfi2|scaffold_1:10181080-10181201 (3.00E-22), respectively. However, after blastn analysis in the GenBank (nr database), similarities were found to the large subunit ribosomal RNA gene from *Brettanomyces custersianus* (6.00E-95 to accession KY106219 for clone 5A10) and the ADP-ribosylation factor 1-like gene from *Musa acuminata* subsp. *malaccensis* (2.00E-149 to accession XM_009405566 for clone 5D2), indicating that these ESTs are from a microorganism present in the biostimulant (*B. custersianus*) and to the banana plant, respectively.

## 2 Experimental design, materials, and methods

### 2.1 Biostimulant conditions

The liquid biostimulant was elaborated at the Research Biotechnological Center of Ecuador (CIBE), by the agricultural technique area. The conditions for the biostimulant elaboration were established by this area according to Jimenez et al. [2], and the final product presented the following parameters: pH between 3.81 and 4.17, conductivity 20.23–24.47 mS/cm, solutes 5.69–7.81 g/L, density 1.03–1.04 g.cm^3^, organics remains 1.89–4.91%, salinity 8.95–10.59 ppm at a temperature of 26.6 °C.

### 2.2 *Pseudocercospora fijiensis* growth conditions

To obtain conidia for pathogen infection, *P. fijiensis* isolates stored at −80 °C in the laboratory were subcultured in Petri dishes with potato dextrose agar medium (PDA) according of the manufacture recommendations. Samples were incubated at room temperature for seven days. The mycelium of the pathogen was isolated directly from the dish and crushed manually. The product was transfer to a modified V8 medium [3] and left at room temperature for 7 days. The conidia obtained were diluted in sterile pure water with Tween™ 20 (0.005%) and the concentration was measured with a Neubauer chamber.

### 2.3 Plant conditions, biostimulant application and pathogen infection

The plants were obtained by *in vitro* propagation and transferred to the greenhouse in substrate in seedbeds after rooting. Plants were maintained for 5–7 weeks (phase I), and then the plants were transferred to individual bags with substrate (phase II) for two weeks to reach two functional leaves. For this assay six biological replicates were used for each treatment (foliar and substrate biostimulant application) and the control (plants without biostimulant application). All the plants were infected with the pathogen. Leaf samples were collected for RNA extraction every two weeks after pathogen inoculation, for a period of two months (four time points in total). Biostimulant application was also performed after each collection time point.

The conidia solution (20,000 conidia/ml) was applied directly to the leaves of banana plants by using an airbrush Badger 100 and the different treatments were maintained separated by aluminium-framed box, covered with transparent plastic according to Sánchez Timm et al. [4].

### 2.4 Total RNA extraction

The RNA extraction was performed by phenol-chloroform method from leaves. Briefly, the chemical lysis of the cells was performed by adding 1 ml of lysis buffer containing NaCl at 400mM, TRIS-HCl at 10mM and EDTA at 2mM, 4% of PVP (MW: 40.000) and 7 μl of β-mercaptoethanol for each 1 ml to a 150–200 mg of plant tissue following of hard vortex immediately. Then, 2% of SDS and 300 μg of proteinase K with and incubation in water bath at 55 °C for 1 hour was performed. This solution was clarified by centrifugation at 12000 RPM at 4 °C for 30 minutes and the supernatant was transferred to a new clean tube. To separate the RNA, an equal volume of phenol:chloroform:isoamylalcohol (25:24:1) was added, following by vortex for 30 seconds. This separation process was repeated twice. An equal volume of chloroform:isoamyl alcohol (24:1) was added, following by vortex and final separation by centrifugation at maximum velocity at 4 °C for 5 minutes. The supernatant was transfer to a new clean tube and added 1:10 (v:v) of sodium acetate at 3 M (5.2 pH) and 2.5:1 (v:v) of chilled ethanol at 96% of purity. Then, the solution was incubated at 4 °C for 2 hours and precipitated by centrifugation of 14.000 RPM at 4 °C for 30 minutes. The supernatant was discarded, and the pellet resuspended in 100 μl of DEPC-treated ultrapure water. Finally, 0.5:1 (v:v) of LiCl at 6 M was added and mix by inverting the tube, following incubation at −20 °C overnight. The precipitation was performed by centrifugation at 12.000 RPM at 4 °C for 30 minutes. The supernatant was discarded, and the pellet was washed by chilled ethanol at 70% of purity. The pellet was resuspended in 50 μl of DEPC-treated water. The concentration and quality were measured by absorbance (Nanodrop™) and agarose gel electrophoresis (1.5% agarose).

### 2.5 SSH libraries preparation and sequencing

Two different libraries were developed. The first one, used the mock-treated plants (control) as the driver, and the treatment with foliar application of biostimulant as the tester. The second library was developed using the mock-treated plants (control) as a driver and the substrate biostimulant application as the tester. The pool of each treatment and control were prepared by mixing six RNA samples (from six biological replicates) for each time point collected, which was performed every 2 weeks after *P. fijiensis* infection for a total of four collection dates (24 samples total for each pool). The libraries were generated by using the suppression subtractive hybridization technique with the PCR-Select™ cDNA Subtraction Kit (Clontech Laboratories, Inc., USA) starting with 20 μg of RNA for each pool.

Once the expressed sequence tags (ESTs) were obtained, fragments were cloned into pGEM®-T Easy Vector System (Promega, USA) and transferred into *Escherichia coli.* The *E. coli* was grown in solid lysogen broth (LB) medium and the size of each EST was analysed by end-point PCR using M13 universal primers directly to the isolated plasmid. The PCR product was separated in agarose gel electrophoresis and only the amplicons with sizes higher than 500 bp were sent for sequencing (Macrogen, USA).

The plasmids were isolated using an alkaline method, starting with over-night culture of *E. coli* in LB medium at 37 °C and 200 RPM. Briefly, cells were collected by centrifugation at maximum velocity for 10 minutes. The bacterium pellet was resuspended in a lysis buffer with glucose (50mM), tris-HCl (25mM, pH8), EDTA (10mM) and RNaseA (20 μg/ml) and incubated on ice for 5 minutes. Then, a second solution was added with NaOH (0.2 N) with SDS (1%) and mixed by inverting the tube, followed an incubation on ice for 5 minutes. To clarify the solution, centrifugation was performed at 13000 RPM for 5 minutes. Then, the upper phase was transfer to clean tubes and an equal volume of isopropanol was added. The mix was centrifuged at 13000 RPM for 10 min and the pellet was washed twice with ethanol at 70% and resuspended in TE buffer (pH 8). The quantity and quality of the plasmids were determined by absorbance (Nanodrop™) and the plasmids were sent for sequencing.
